# Electroconvulsive therapy for Depression in Anorexia Nervosa. A review

**DOI:** 10.1192/j.eurpsy.2022.1906

**Published:** 2022-09-01

**Authors:** F. Azevedo, R. André, I. Donas-Boto, D. Jeremias, C. Almeida

**Affiliations:** 1 Centro Hospitalar de Lisboa Ocidental, Psychiatry, Lisbon, Portugal; 2 Centro Hospitalar Universitário Lisboa Norte, Psychiatry, Lisboa, Portugal; 3 Centro Hospitalar de Lisboa Ocidental, Psychiatry Department, Lisboa, Portugal; 4 NOVA Medical School, Psychiatry And Mental Health, Lisboa, Portugal

**Keywords:** ECT, Anorexia, Electroconvulsive therapy, Anorexia nervosa

## Abstract

**Introduction:**

Anorexia nervosa has an important burden on both patients and families, with important comorbidities such as depression and obsessive symptoms. These are more resistant to pharmacological treatment than in non-anorexia patients, due to both biological and psychological mechanisms. Electroconvulsive therapy is the best available therapy for treatment resistant depression making it a treatment to consider in treatment resistant depression in anorexia though only case reports exist.

**Objectives:**

To review the current evidence for electroconvulsive therapy of depression in patients with anorexia nervosa as well as it’s ethical challenges

**Methods:**

Non-systematic review of the literature with selection of scientific articles published in the past 10 years; by searching Pubmed and Medscape databases using the combination of MeSH descriptors. The following MeSH terms were used: “electroconvulsive therapy”, “anorexia nervosa”.

**Results:**

Electroconvulsive therapy in anorexia has no controlled trials with mostly case reports available on scientific databases. It presents important challenges due to patient age, medical status and ethical challenges. Even less evidence exist for electroconvulsive therapy in children and adolescents than for adults, anorexia can complicate medical status presenting an anesthetic and life-support challenge and it’s egosyntonicity can place a legal and ethical challenge when patient refuses treatment.

**Conclusions:**

Anorexia has a dramatic burden on patients and families affected, with integrated evidence-based treatment being necessary both for treating the current episode and for remission prevention. Case-reports show that electroconvulsive therapy can play a role on treatment resistant depression in anorexia.

**Disclosure:**

No significant relationships.

